# malERA: An updated research agenda for health systems and policy research in malaria elimination and eradication

**DOI:** 10.1371/journal.pmed.1002454

**Published:** 2017-11-30

**Authors:** The malERA Refresh Consultative Panel on Health Systems and Policy Research

## Abstract

Health systems underpin disease elimination and eradication programmes. In an elimination and eradication context, innovative research approaches are needed across health systems to assess readiness for programme reorientation, mitigate any decreases in effectiveness of interventions (‘effectiveness decay’), and respond to dynamic and changing needs. The malaria eradication research agenda (malERA) Refresh consultative process for the Panel on Health Systems and Policy Research identifies opportunities to build health systems evidence and the tools needed to eliminate malaria from different zones, countries, and regions and to eradicate it globally. The research questions are organised as a portfolio that global health practitioners, researchers, and funders can identify with and support. This supports the promotion of an actionable and more cohesive approach to building the evidence base for scaled-up implementation of findings. Gaps and opportunities discussed in the paper include delivery strategies to meet the changing dynamics of needs of individuals, environments, and malaria programme successes; mechanisms and approaches to best support accelerated policy and financial responsiveness at national and global level to ensure timely response to evidence and needs, including in crisis situations; and systems’ readiness tools and decision-support systems.

## Abstract 2

Marcel Tanner and colleagues examine progress in health systems and policy research for malaria elimination and eradication.

**Summary points**

- Since 2011, few research questions identified in malERA Health Systems and Operational Research agenda have been addressed, or only addressed in a fragmented way by scientists and implementation researchers at national and international levels. Multiple factors, including funding, dependency upon existing national and provincial health systems to deliver, and limited buy-in by a range of disciplines into the malaria agenda, have contributed to this limited uptake.
- To address the complexity of health systems and any changes or adaptations within them requires a systemic and transdisciplinary approach in the conceptualization and conduct of the research. Transdisciplinary research requires the inclusion of various sectors and agencies, communities, and civil society, as well as inclusion and integration of a range of research disciplines including policy, management, and social sciences.
- Presenting the research and development agenda here as a portfolio ensures the transdisciplinary and multi-stakeholder approach and can therefore help funders and researchers take action and engage in the pursuit of this agenda according to their own diverse priorities.

## Introduction

Between 2000 and 2015, a major expansion of WHO-recommended interventions have contributed to a 58% reduction in the global malaria mortality rate (69% among children under 5 years old in Africa), resulting in an estimated 6.2 million lives being saved from a malaria-related death [1].

As part of the initial malaria eradication research agenda (malERA) process, published in 2011, a consultative group on health systems and operational research established a list of research priorities presented in a matrix system organized by the different levels (community, facility, district, and national levels) and building blocks of the health system [2]. Health-system building blocks are described in Box 1. The health systems concept and framework was based on the guiding summary on ‘health system thinking’ perspective formulated by the Alliance for Health Policy and System Research [3]. One key gap that was identified in 2011 was a tool to diagnose impediments in a given health system that limited the effective and equitable impact of malaria interventions [2].

**Box 1. Health system building blocks that together constitute a complete system.**

- Governance: Ensuring strategic policy frameworks combined with effective oversight, coalition building, accountability, regulations, incentives, and attention to system design.
- Human resources: Responsive, fair, and efficient, given available resources and circumstances, and available in sufficient numbers.
- Financing: Raising adequate funds for health in ways that ensure people can use needed services and are protected from financial catastrophe or impoverishment associated with having to pay for them.
- Health information: Ensuring the production, analysis, dissemination, and use of reliable and timely information on health determinants, health systems performance, and health status.
- Service delivery: Including effective, safe, and quality personal and nonpersonal health interventions that are provided to those in need, when and where needed (including infrastructure), with a minimal waste of resources.
- Commodities: Including medical products, vaccines, and other technologies of assured quality, safety, efficacy, and cost-effectiveness, and their scientifically sound and cost-effective use.

The momentum created by the successes since the turn of the century has led to reiterated commitment and partnership and 2 complementary key documents, (i) the Global Technical Strategy (GTS) for malaria 2016–2030 [4] and (ii) the Action and Investment to Defeat Malaria (AIM) 2016–2030 [5], which were launched respectively by the Global Malaria Programme (GMP) of WHO and the Roll Back Malaria (RBM) Partnership in 2015. The GTS and the investment and advocacy framework partnership document, AIM, pave the way for intensifying malaria control and elimination efforts and set ambitious yet realistic targets and goals. The GTS clearly outlines a common technical strategy while AIM provides the investment framework to reach these technical targets. Moving forward, GTS and AIM need to be brought to the country level and be clearly reflected in the national malaria control and elimination strategic plans, as well as in the overall national health policy and strategy.

In view of these advances, many challenges need to be coherently addressed in order to not threaten continued progress. These challenges are as reflected in GTS and AIM:

1. Emerging parasite resistance to antimalarial medicines and mosquito resistance to insecticides.
2. Systemic and technical obstacles, such as the inherent weakness of health systems, including poor disease surveillance and limited pharmaceutical regulation.
3. Lack of adequate technical and human resource capacities including community engagement.
4. High prevalence of asymptomatic infections and unknown dimension of the existing asymptomatic reservoir.
5. Diversity of vectors and their behaviour.

To complement GTS and AIM, a systematic review of progress in research and development (R&D) and a consultative process to update the research agenda, ‘malERA Refresh’, was launched [6]. malERA Refresh can be seen as the third pillar, defining research priorities to support the GTS targets, while the business case for achieving the GTS targets is provided by AIM.

### Scope of this report

In the refresh of malERA presented here, an expert panel focussed on health systems and policy research reviewed the progress made since 2011 and set out an updated agenda and research portfolio necessary to support the global malaria elimination agenda. While addressing broader health system development needs would be beneficial to this as well as other public health agendas, it was not the focus of this piece of work, and others have addressed these in detail. A particular emphasis was placed on the elimination phase in order to best accompany the GTS and the AIM framework, as well as to assist the national strategies and operational plans. The main differences between the 2011 and 2017 health system agendas are that this 2017 agenda:

- Focusses on malaria, in particular malaria elimination. The 2011 agenda was a broader view on health systems and all phases of control to elimination.
- Recognizes that 1 country can have various phases of control to elimination occurring within its programme, so does not, as was done in 2011, group countries into phases.
- Frames the agenda as a portfolio from hypothesis driven (research), development of tools or interventions (R&D), or synthesis of existing information and evidence (evaluation science).
- Recognizes the need to tailor questions to different settings and therefore has left the questions deliberately broad to allow general application to specific settings.

### Context and rationale

In spite of increased financial and commodity resources, progress in malaria control and elimination in most countries has been slower than expected. Among the main reasons for the slow pace are constraints on the delivery of essential health interventions, including malaria interventions, at sufficient levels of coverage and quality to populations in need. At the same time, the attainment of GTS targets and goals, within the AIM investment framework, will rely heavily on well-performing health systems for the sustained control and elimination phases.

Progress has been made in recent years towards better understanding health systems and how to strengthen them. The previous malERA health systems working group recommendations have contributed to that change, which is clearly reflected in AIM as well as health-systems thinking and is acting as an integral part of WHO GMP Malaria Policy Advisory Committee (MPAC) recommendations [7]. Moreover, global health initiatives have certainly increased funding for strengthening national health systems to accelerate progress on universal access to essential health interventions, particularly for HIV/AIDS, tuberculosis, malaria, and immunisation. Initiatives such as the Task Force on Innovative Financing for Health Systems [8] testify to the increased commitment to funding health systems and momentum in favour of health system strengthening, minimising the negative impacts of vertical programmes on health systems, and leveraging malaria activities through other health programmes.

However, significant work and questions remain. By systematically reviewing the literature, as well as ongoing research projects in health systems in the Malaria Eradication Scientific Alliance (MESA) Track database [9] (see Methods and approaches section), the panel concluded that many research priorities identified in the 2011 Panel on Health Systems and Operational Research have hardly—or only in a fragmented way—been picked up by scientists and implementation researchers at national and international levels.

The malERA portfolio dealing with health systems attracted insufficient funding and interest over the last few years. One possibility for this is that funding for malaria is more vertically targeted on parasites, diagnostics, treatment, and vectors than on broad health systems and in part because the agenda was more accessed by malaria scientists and not the broader health system research community and public health practitioners. Additionally, many of the disciplines required to engage in the health system agenda are not presently majorly involved, and the existing malaria scientific community are not well equipped to implement the health system agenda. There is a need to reiterate the agenda as established in 2011, but more importantly—by building on it—add new dimensions to the research and R&D needs in the field of health systems and policy research. This paper presents the agenda as a portfolio ranging from priorities in evaluation science to specific research questions to R&D issues (see Box 2 for definitions).

**Box 2. malERA Refresh research categories to support a portfolio approach to health systems and policy research.**

- Evaluation science: Where information from the field and across several sites, contexts, and case studies already exists (mainly WHO/University of California San Francisco [UCSF] elimination case studies, RBM Progress and Impact reports, etc.) and an answer to the research question could be obtained by comparative, synthetic analysis.
- Research: Where new information is needed to answer a research question based on an underlying hypothesis or where new hypotheses need to be tested.
- R&D: Where an R&D process is required, based on underlying hypotheses, to develop a tool, a tool kit, or new approaches (e.g., surveillance as an intervention, ‘surveillance-response’). These tools or approaches would be created based on available data and information to serve implementers in carrying out their work. The development of this tool, tool kit, or approach becomes the activity pertinent to this portfolio.

**Fig 1:**
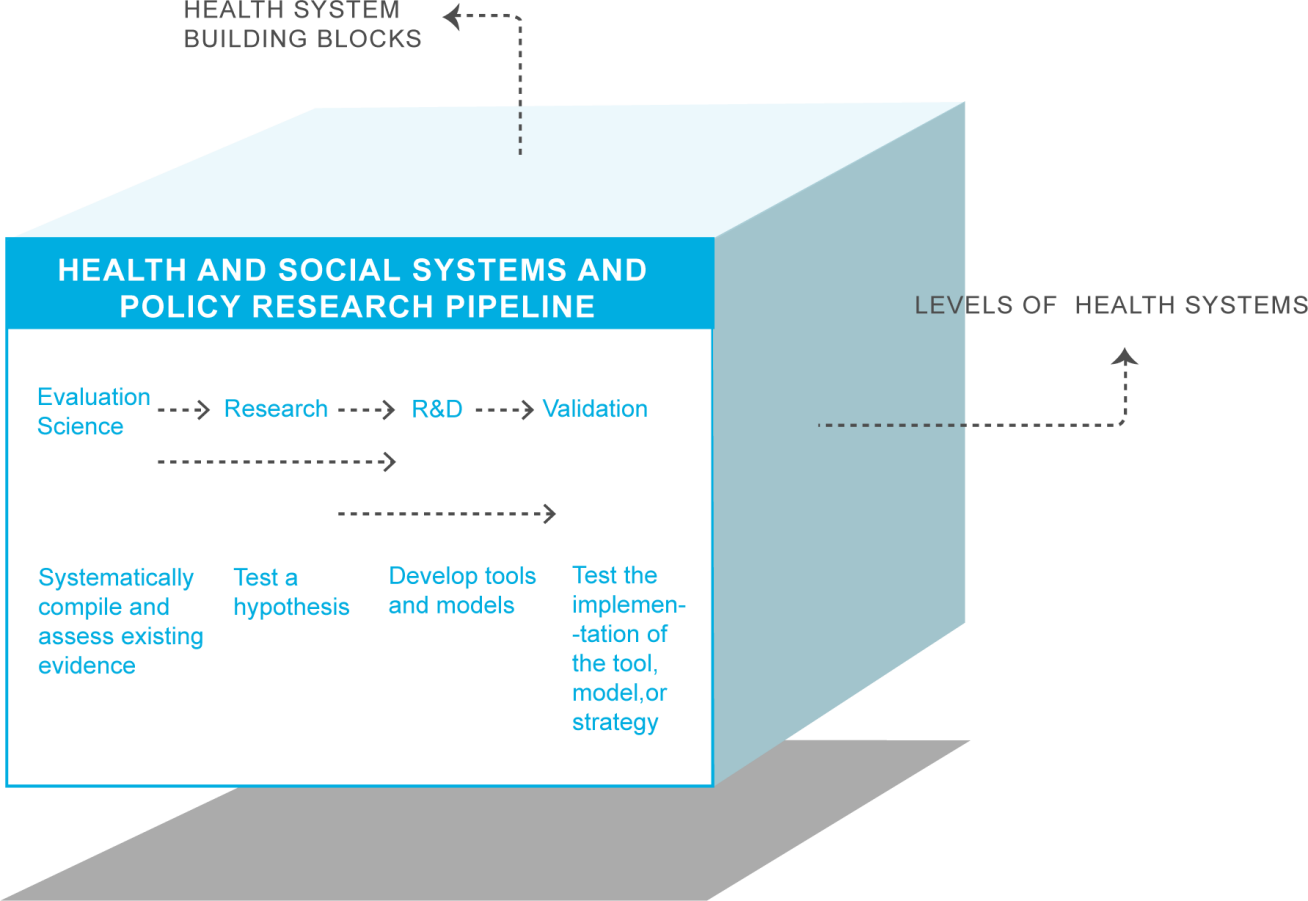
Research agenda cube for health systems and policy research. R&D, research and development.

Funding partners and implementation groups have clearly prioritized the quick delivery of malaria interventions and a system of monitoring and evaluation (M&E) linked to their funding and not necessarily integrated into routine health systems M&E, including health systems research. Contrasting this to governments in countries affected by malaria, which are tasked with responding to ambitious global targets and goals, reveals that these countries have long recognised the need for health system thinking and research in order to meet the healthcare needs of their populations. As noted above, addressing the more challenging but also more sustainable issue of health system strengthening to support malaria eradication tends to be postponed as it is still seen, conceptually and operationally, as a daunting issue. This difficulty can be overcome by presenting the research and R&D priorities within a portfolio approach tailored to the different levels and building blocks of a given health and social system (Fig 1). Addressing the list of key research questions outlined in this paper would go a long way in strengthening malaria control and elimination and inscribing it into the fundamental health infrastructure of endemic countries (full portfolio of questions in Table 1).

**Table 1:** Priority questions for evaluation science, research, and R&D in health systems and policy research.

**Evaluation science**	**Health system building block**	**Health system level**
1st Priority: **What are the social & political drivers that influence malaria elimination success within and across regions at national and regional levels?**	Governance	National
2nd Priority: **How do optimized delivery strategies meet changing and dynamic needs of the system requirements and/or community perceived needs?**	Service delivery	Community
2nd Priority: **What are effective strategies and tools to sustain community workers’ engagement in malaria activities during intensive control and elimination?**	Human resources	Community
Additional Emphasis: **Evaluation of case studies (including the malaria elimination case study series) regarding the determinants for successful scale-up of district management, financing, and human resource models in different contexts and settings.**	Cross-cutting	Facility
What mechanisms support effective integration of communicable disease surveillance?	Information	All
With an emphasis on decentralization, what are the management strengths required, and how can the readiness of the different health systems structures be assessed for malaria elimination in different settings?	Governance	All
What is the range of effective HIS and tools to capture and use information at the community level?	Information	Community
What mechanisms, tools, and strategies can be utilized to sustain active community engagement in intensive malaria control and elimination?	Governance	Community
What is the range of HIS and tools to effectively capture and use information at the community level?	Information	Community
How to scale up measures to ensure quality and quantity of health commodities at the community level in both public and private sectors, especially to remote and vulnerable populations?	Information	Community
What are the key essential service delivery tools implemented at PHC level to ensure quality of malaria elimination activities (prevention, treatment, and surveillance-response) at facility and community levels in different system contexts?	Service delivery	Community
What are the key essential service delivery tools implemented at PHC level to ensure quality of malaria elimination activities (prevention, treatment, and surveillance-response) at facility and community levels in different system contexts?	Service delivery	Facility
What is the range of effective HIS and tools to capture and use information at the national level?	Information	National
Comparatively assess across countries and settings which mechanisms best support accelerated policy and financial responsiveness at the national level to ensure timely response to evidence and needs, including in crisis situations.	Governance	National
Comparatively assess across various countries and subnational settings which are the effective approaches and their determinants to transition funding to sustainable financing sources.	Financing	National
What are efficient and ethical approaches to health security issues that can be applied to managing malaria in epidemics, reintroduction, and resurgence?	Governance	National
Comparatively assess which mechanisms best support accelerated policy and financial responsiveness at the global level to ensure a timely response to evidence and needs, including in crisis situations.	Governance	Regional/Global
What are the social and political drivers to influence malaria elimination within and across regions at country and regional levels?	Governance	Regional/Global
**Research**	**Health system building block**	**Health system level**
1st Priority: **What are the decision-making frameworks required to eliminate and prevent reestablishment of malaria?**	Governance	National
2nd Priority: **What is the best way to optimize malaria elimination delivery strategies to meet the changing dynamics of needs of individuals, environments, and malaria programme successes?**	Service delivery	Community
What are the determinants of efficiency of community-level health service delivery and of community systems, with an emphasis on malaria elimination outcomes?	Financing	Community
Which innovative measures would improve quality and quantity of malaria commodities at the community level in both public and private, especially to remote and vulnerable populations for malaria elimination?	Commodities	Community
What management tools and structures can improve transparency, accountability, and effectiveness of health facilities for malaria elimination activities (coverage, equity, and quality) in different contexts and systems?	Governance	District
What mechanisms and approaches best support accelerated policy and financial responsiveness at the national level to ensure timely response to evidence and needs, including in crisis situations?	Governance	National
In the context of a shared public health target of malaria elimination, what are the determinants and the effective modes & models of intercountry & cross-border collaboration for policy & implementation?	Governance	National
What adaptive changes are needed in operations (management, financing, human resources, and responsibilities) of health systems to move to and support malaria elimination?	Cross-cutting	National
What are effective models of government leadership at leveraging integrated activities cross-sectorially for malaria elimination?	Governance	National
What ensures effective governance and accountability to support elimination?	Governance	National
What enables ownership of elimination at national and regional levels?	Governance	National
What are effective mechanisms to leverage financing for malaria prevention from health insurance schemes?	Financing	National
What are effective mechanisms and approaches to transition from external funding to sustainable financing sources?	Financing	National
What mechanisms and approaches best support accelerated policy and financial responsiveness at the global level to ensure timely response to evidence and needs, including in crisis situations?	Governance	Regional/Global
In the context of a shared public health target of malaria elimination, what are the determinants and the effective modes and models of intercountry and cross-border collaboration for policy and implementation?	Governance	Regional/Global
What are the decision-making frameworks required to eliminate and prevent reestablishment?	Governance	Regional/Global
What adaptive changes are needed in operations (management, financing, human resources, and responsibilities) of health systems to move to and support malaria elimination?	Cross-cutting	Regional/Global
What are effective models of government leadership at leveraging integrated activities cross-sectorially for malaria elimination?	Governance	Regional/Global
What ensures effective governance and accountability to support elimination?	Governance	Regional/Global
What enables ownership of elimination at national and regional levels?	Governance	Regional/Global
**R&D**	**Health system building block**	**Health system level**
1st Priority: **What are the health planning and funding models and tools required to eliminate and prevent reestablishment of malaria?**	Cross-cutting	National
2nd Priority: **What tools (existing, new, or a combination of both) can measure systems’ readiness at community, facility, and district levels in an integrated way to support elimination and prevention of reintroduction?**	Cross-cutting	All
Develop the tools and SOPs for effectiveness decay analyses.	Cross-cutting	All levels
Development of tools and strategies to strengthen and sustain active community engagement in intensive control and malaria elimination.	Cross-cutting	Community
How can community components of integrated service delivery approaches (IMCI, IMAI, and ICCM) be adapted to malaria elimination and prevention of reintroduction?	Governance	Community
Develop a broad system readiness tool to include stop/start decisions, appropriate economic tool and approaches (e.g., CEA and CBA), and link the tool to decision-support systems.	Service delivery	National
What are the health planning and funding models and tools required to eliminate and prevent reestablishment of malaria?	Cross-cutting	Regional/Global
Develop a broad system readiness tool to include stop/start decisions, appropriate economic tool and approaches (e.g., CEA and CBA), and link the tool to decision-support systems at the regional level.	Cross-cutting	Regional/Global

CBA, cost-benefit analysis; CEA, cost-effectiveness analysis; HIS, health information system; ICCM, intergrated community case management; IMAI, integrated management of adolescent and adult illnesses; IMCI, integrated management of childhood illnesses; PHC, primary health care; R&D, research and development; SOP, standard operating procedure

## Methods and approaches

In 2009, WHO [3] defined health systems research as ‘the purposeful generation of knowledge that enables societies to organize themselves to improve health outcomes and services.’ It is concerned with how health services are financed, delivered, and organised and how these functions are linked within an overall health and social system with its associated policies and institutions.

To understand and address the complexity of health systems and implementation of change into those systems requires not only a systemic but also clearly a transdisciplinary approach. Transdisciplinary research entails approaching a complex problem in ‘a way that can (a) grasp the complexity of problems, (b) take into account the diversity of life-world and scientific perceptions of problems, (c) link abstract and case-specific knowledge, and (d) constitute knowledge and practices that promote what is perceived to be the common good’ [10]. This approach to research requires the inclusion of various sectors and agencies, community, and civil society, as well as inclusion and integration of a range of research disciplines including policy and political science, management, economics, and social sciences (including anthropology, sociology, and development sciences).

Based on this context, the review of the research agenda was pursued in 2 steps, each step engaging the panel members as described in S1 Text, to organise the health systems research needs in malaria elimination as a portfolio of evaluation science, research, and R&D. This kind of portfolio approach should make it easier for researchers and funding agencies to identify their subjects of interest. The panel used the modified Nominal Group Approach to identify the priority questions in each category [11] (see S1 Text).

## Results: Fundamentals and prerequisites

### Changing focus from pilot studies to scale-up

Based on analysis of MESA Track and extensive literature search (S1 Text) and taking into account the time it takes for the first malERA process to gain traction, it emerged that research taken up after the first malERA process led to a few interesting pilot studies. Even when conclusive, these projects remained fragmented and rarely led to or indicated initiation of scaled-up implementation of the key findings and recommendations. This malERA Refresh process aims to avoid fragmentation and move beyond pilot studies.

When research brings forward conclusive results and recommendations, a strong effort should be placed on scaling them up in that context. When results have not been internally and externally validated (e.g., because of the small size of pilot studies or fragmentation of research), then more research and evaluation efforts should focus on gathering the information pertinent to inform the scale-up for impact of interventions [8].

### Effectiveness decay

**Fig 2:**
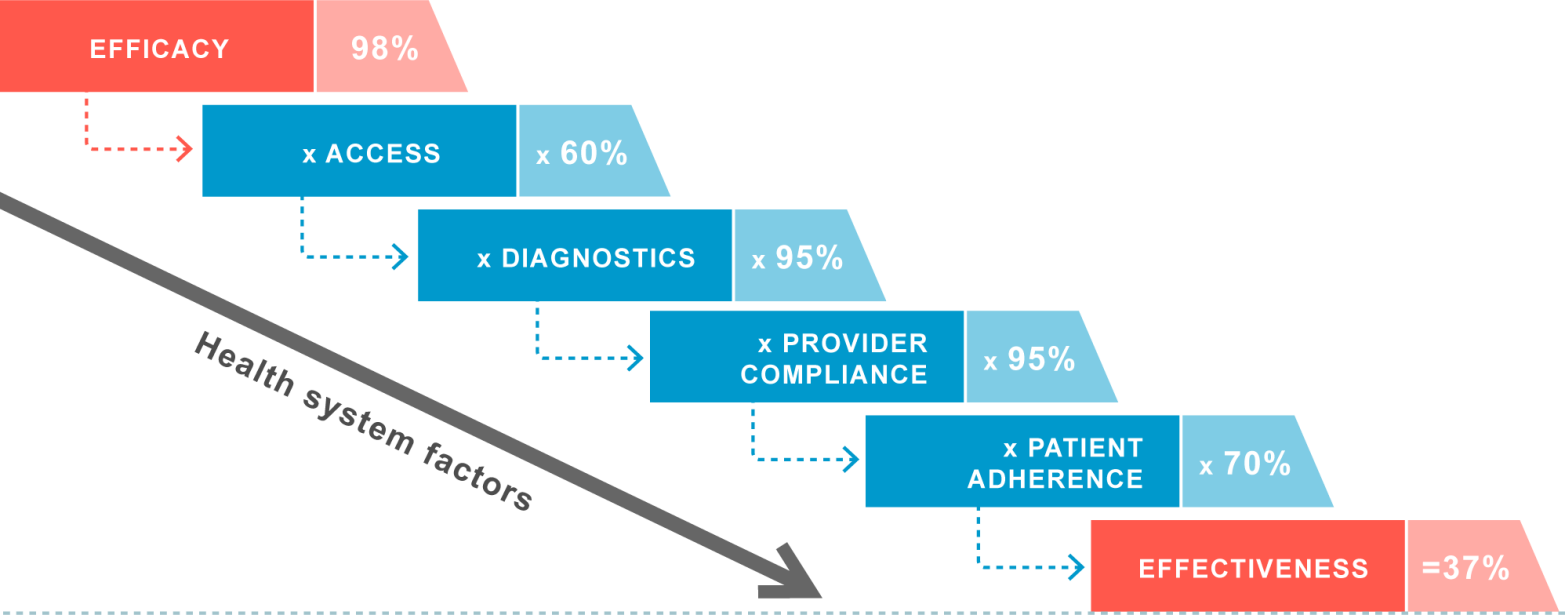
Effectiveness decay. Loss of effectiveness of interventions within the health system is depicted here by steps. The pattern of effectiveness decay (how much is lost and at what step) varies and depends on the specifics of a given health system [12]. The percentages of decay/loss are hypothetical.

Effectiveness of interventions is lost at almost all (if not all) levels of the health system. This means that at 1 or several points in a health system, efficacious interventions may lose some of their effect because they cannot be applied or implemented effectively; this is called effectiveness decay. By analysing effectiveness decay within a specific health and social system one can elucidate at which levels the greatest loss of effectiveness are occurring (Fig 2) and then test strategies with which to intervene and recover effectiveness of the intervention. For malaria control and elimination, understanding the equity effectiveness of interventions, that is whether all who need to access and use the intervention can do so equally, is of particular importance.

The effective execution of the elimination agenda would benefit from high performance of health systems that can deliver the optimal combination of malaria interventions at high and equitable levels of quality and population coverage. This requires the concerted and combined strength of all the health system building blocks. Program managers need to be able to detect the reasons why coverage levels are inadequate or inequitable and, based upon that, develop appropriate ways to address these reasons within their health and/or social system. And for system interventions and strengthening to be effective and efficient, they need to be able to diagnose those problems and their determinants and interactions (decay at each level is not the same in all health systems). The system effectiveness analytic framework has proven very useful as a health system diagnostic tool to identify areas where and how system factors need to be and can be strengthened. This framework sees a cascade of progressive loss of intervention efficacy (Fig 2) and addresses it through system-specific issues of access, targeting, provider compliance, and client adherence as the system tries to deliver an intervention at effective coverage levels in real-world settings. Many programmes have used it to analyse the determinants of coverage but results are often yet to be translated into targeted health systems. Such an analysis will need to be complemented with an analysis of allocation efficiency, which will help to optimize a portfolio of multiple interventions in a given context to maximize health impact.

### Programme efficiency and health system readiness

After the effectiveness decay analysis, programmes at various levels within a country (local, subnational, and national) can establish the most efficient operational mix to meet their targets and objectives. They need to precisely define when and where interventions need to start and stop and what time frame is involved for certain operations. Based on surveillance data and prevalence and transmission intensity, and assisted by modelling, interventions or mixes of interventions to be applied in an integrated way should be clearly defined [13]. Starting from the initial decay analysis at the national and subnational levels, it becomes a health systems surveillance tool to be part of the national programme and the surveillance-response approaches. By ignoring the continued M&E of the health systems effectiveness, control and elimination programmes are at risk of inefficiencies and unrecognized loss of effectiveness.

## Results: Research and R&D portfolio

Examples of the research portfolio discussed by the consultative panel are presented in Box 3, starting with cross-cutting priorities relevant to all health systems and policy research topics and then going over the research agenda in evaluation science, research, and R&D of new tools categories. For each research question in each category, the health system levels and the building blocks are mentioned. The top 2 priority questions and the question on which the panel chose to place special emphasis are always listed first in each category. A rationale is provided in the portfolio to offer some background and context on the issues raised. Then follows a list of additional questions linked to the priority questions to suggest key questions also to be answered.

**Box 3. Examples of research questions in the research agenda for health systems and policy research.**

**Evaluation science (synthetic**, **comparative evaluation of existing interventions and case studies)**

- What were the social, regulatory, and political drivers that supported or affected the ability of malaria elimination systems requirements to be integrated into existing health system in the country/countries that have eliminated malaria?
- What approaches to engaging community health workers in malaria elimination activities support sustaining their active engagement in service delivery in the country/countries that have eliminated malaria?
- What were the cost-effective strategies for optimal delivery of various components of a malaria elimination programme to targeted populations/locations developed in the country/countries that have eliminated malaria?
- What role has housing and rural/urban environmental improvements played in supporting malaria elimination and prevention of reintroduction in countries that have achieved malaria elimination?

**Research** **(test hypotheses)**

- That the integration of malaria elimination surveillance and response approaches can be cost-effectively integrated into other infectious disease surveillance systems.
- That communities can play an effective role in active efforts at transmission reduction (as opposed to reducing morbidity and mortality from malaria)?
- That routine primary healthcare services including maternal and child health services can sustain access to and utilization of individual and household-level malaria interventions to sustain malaria elimination and prevent reintroduction of malaria.
- That improved methods of data collection, synthesis, visualization, and real-time availability will increase the timeliness and effectiveness of health workers’ responses to malaria cases in elimination settings.

**R&D (develop and test tools**, **approaches/strategies, and models)**

- Development of IMCI and IMAI updated with new diagnostic tools and adapted to the malaria elimination context.
- Development and scaling-up of tools to measure systems readiness for malaria elimination and prevention of reintroduction at local and subnational levels.
- Development of mechanisms to support and maintain financial and political responsiveness to malaria elimination and prevention of reintroduction.
- What are the messages and best means of conveying these to various communities/at risk populations to support access, acceptability, and utilisation of malaria interventions nearing elimination?

**Fig 3:**
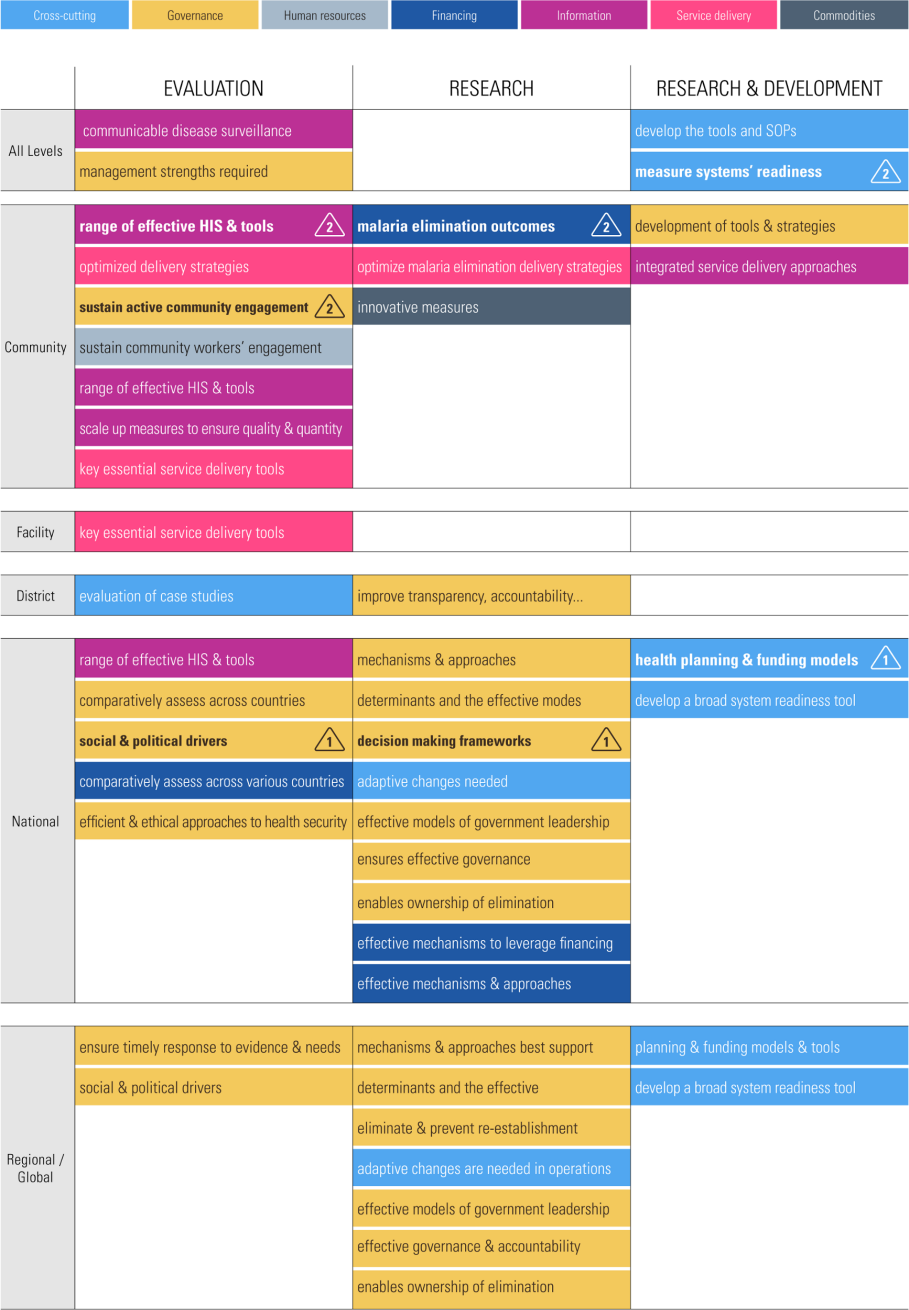
Overview of health systems and policy research portfolio. HIS, health information system; SOP, standard operating procedure.

A synthetic presentation of the full research portfolio is presented in this paper (Fig 3), color-coded for areas of activities: cross-cutting, governance, human resources, financing, information, service delivery, and commodities. Questions are ranked according to the health system level to which they pertain: all levels, community, facility, district, national, and regional/global. Keywords are used to refer to questions detailed later in the document. Following the synthetic table, the list of research questions is presented for evaluation science, research, and R&D, starting with priority questions (Table 1).

### Capacity building and training

Capacity building and training is a priority cross-cutting all areas emphasized in this document. No positive outcome can be anticipated if an appropriately trained and competent work force cannot be relied upon. This is indeed an absolute prerequisite for well-functioning health systems and delivery of malaria control and elimination interventions.

### Surveillance and M&E

A theme common to all programmes and systems areas is the critical role that surveillance and M&E play in every phase. Adequate surveillance and M&E methods must be in place to monitor effectiveness and inform the decision-making process. Surveillance and M&E fall under the ‘surveillance-response’ umbrella, where essential data are collected in space and time to inform well-tailored, integrated-response actions.

### Supply-chain strengthening

A very common cause of loss of malaria programme effectiveness is the disruption in appropriate stocks of life-saving commodities for at-risk populations. No malaria control or elimination programme can hope to achieve its objectives without addressing the issue of stock outs of commodities and strengthening the supply chain so that an uninterrupted flow of necessary quality tools can be available at all times for people who most need them.

### Community involvement and engagement

A critical element of success and of sustainability of any intervention, and particularly malaria elimination interventions, is community ownership and engagement. The RBM AIM (2015) noted as countries move along the path to elimination, resource requirements, processes, and services change, requiring national systems to adapt and improve, and to deepen their level of community engagement and in Chapter 6 discussed this in more detail, especially the role of people-centred and participatory research design and approaches [5].

## Keeping a dynamic portfolio: Monitoring progress and dissemination of results

Multiple factors have slowed progress in the health systems and operations research agenda since the first malERA initiative, including funding, dependency upon existing national and provincial health systems to deliver, and limited buy-in by a range of disciplines into the malaria agenda. All of these factors have contributed to this limited uptake. The panel felt that in order to keep a dynamic portfolio and not lose traction again, monitoring the uptake of research priorities established during the malERA Refresh process was essential.

The following processes could ensure proper monitoring of research activities: First, MESA, through the established tool of MESA Track and linking up with all research and implementation institutions involved, could be tasked with producing regular updates on progress accomplished [9]. The second important feedback would be from countries (national malaria control programmes level) reporting on evaluation science, research, and R&D tools development. A third approach is to use networks emerging throughout regions such as Asia Pacific Malaria Elimination Network (APMEN) [14] and the 8 eliminating southern African countries (Elimination 8 [E8]) [15] to both socialise and support the implementation of this research agenda, as well as provide platforms for dissemination of lessons learned from the research. This can use innovative platforms like case studies, focussed study tours, peer-to-peer mentoring, workshops, and health system fellowships.

Information collected through these 2 tracks would be regularly reviewed by the malERA Refresh committee and compared across the whole malERA spectrum. The results, regularly published, in combination with updates from national programmes and their partners on status of national research and implementation, would be included each year in the WHO World Malaria Report. Other channels for dissemination and garnering of scale-up support would be through platforms such as the political alliances of African Leaders Malaria Alliance and the Asia Pacific and Leaders Malaria Alliance [16,17]. The malERA Refresh has given and will continue to give the malaria community an opportunity to highlight perhaps the most important gaps in getting the world to the malaria-free goal; namely, the continuous and timely improvement in the operational delivery of interventions through health systems improvement.

## Discussion

Setting health research priorities does not automatically guarantee uptake and funding, though there are examples of how it does [18]. And these health systems issues are ‘wicked’ problems for which no one solution will be found; solutions are not discrete from other systems’ issues, and solutions cannot be divorced from socio-historical-political environments [19]. This agenda will also need a broad range of disciplines to engage in the implementation of the agenda beyond the ‘malaria research community’ to include social and behavioural sciences; political, management, and organisation science; health economics; and health systems specialists. Clearly, this health systems agenda to facilitate malaria eradication entails a transdisciplinary and multi-stakeholder approach and therefore needs to be more broadly disseminated and socialised in these scientific communities, and the findings from this research need to be more broadly disseminated to inform other health system interventions and their implementation, as well as health system strengthening in general.

As discussed in the section ‘Keeping a dynamic portfolio’, there is a need to rigorously provide evidence of the outcomes of the research against the overall eradication agenda. This evidence can attract more attention to and focus on the prioritised agenda for other researchers and funders. The research agenda links to GTS, the strategy, and AIM, the investment framework for action, and creates the essential third pillar on our journey to achieve elimination and eradication. Building the capacity of health and other staff and researchers in malarious countries to undertake health systems research, such as through the WHO TDR Structured Operational Research and Training IniTiative (SORT-IT) approach, is an important step, as many of these research topics need the ‘localisation’ required to understand the impact of different settings and health and social systems upon interventions and their effective implementation. Funding for these sorts of localised studies will likely need to be sourced from national budgets and/or small research grant schemes; be embedded into the M&E budgets of projects, donors, and governments; and/or be part of the university training and research agendas. The larger number of ‘case studies’ can then allow some comparative analyses and synthesis through evaluation science to be undertaken at a larger scale to better identify enablers, barriers, approaches, and tools for others to ‘trial’.

## Conclusion

This remains an ambitious but essential research agenda for malaria eradication. Every tool or intervention developed, for vectors, parasites, drug administration, surveillance and response, etc., needs a health and social system operating in a socio-political system to deliver the intervention effectively. To address challenges relating to health systems, transdisciplinary and multi-stakeholder approaches need to be tested. Results from evaluation science, research and R&D in this field will likely generate multiple nuanced answers. This process will ensure that the research questions put forward in a portfolio approach can be tailored to any given setting, as one size will not fit all, and finally that the maximum efficacy of interventions is maintained universally across the different social, political, and economic differentials for populations to achieve malaria elimination and, finally, eradication.

## Supporting information

S1 Text Further details on panel methodology and prioritization process. (DOCX)

## Abbreviations

AIM: Action and Investment to Defeat Malaria
APMEN: Asia Pacific Malaria Elimination Network
E8: Elimination 8
GMP: Global Malaria Programme
GTS: Global Technical Strategy
HIS: Health Information System
IMAI: integrated management of adolescent and adult illnesses
IMCI: integrated management of childhood illnesses
malERA: malaria eradication research agenda
M&E: monitoring and evaluation
MESA: Malaria Eradication Scientific Alliance
MPAC: Malaria Policy Advisory Committee
RBM: Roll Back Malaria
R&D: research and development
SORT-IT: Structured Operational Research and Training IniTiative

## References

[1] WHO. WHO World Malaria Report 2015. Geneva, Switzerland; 2015.

[2] The malERA Consultative Group on Health Systems and Operational Research. A research agenda for malaria eradication: health systems and operational research. PLoS Med. 2011;8(1):e1000397 doi: 10.1371/journal.pmed.1000397 21311588 10.1371/journal.pmed.1000397 PMC3026705

[3] Research WAfHPaS. Systems Thinking for Health Systems Strengthening. 2009.

[4] WHO. Global Technical Strategy for malaria 2016–2030 WHO. Geneva, Switzerland; 2015.

[5] WHO. Action and Investment to Defeat Malaria 2016–2030. 2015.

[6] Malaria Eradication Scientific MESA. Available from: http://www.malariaeradication.org/. Cited 29 Sept 2017.

[7] WHO. Malaria Policy Advisory Committee Available from: http://www.who.int/malaria/mpac/en/. Cited 29 Sept 2017.

[8] WHO. Scaling up research and learning for health systems: now is the time. WHO, Geneva; 2008. 10.1016/S0140-6736(08)61634-7 18984175

[9] MESA Track database: Malaria Eradication Scientific Alliance (MESA); [Database]. Available from: http://www.malariaeradication.org/mesa-track. Cited 29 Sept 2017.

[10] Hadorn GH, Pohl C. Principles for Designing Transdisciplinary Research. Munic, Germany: oekom verlag; 2007.

[11] Delbecq AL, Van deVEn AH. A Group Process Model for Problem Identification and Program Planning. Journal of Applied Behavioral Science. 1971;7:466–92.

[12] Vlassoff C, Tanner M. The relevance of rapid assessment to health research and interventions. Health Policy and Planning. 1992;7(1):1–9.

[13] The malERA Refresh Consultative Panel on Combination Interventions and Modelling. malERA: An updated research agenda for combination interventions and modelling in malaria elimination and eradication. PLoS Med. 2017;14(11):e1002453 https://doi.org/10.1371/journal.pmed.1002453 10.1371/journal.pmed.1002453 PMC5708628 29190295

[14] Whittaker M, Smith C, Mouzin E. The Asia Pacific Malaria Elimination Network: Supporting the common goal of a malaria free Asia Pacific. Geneva, Switzerland; 2014.

[15] E8, the eight eliminating southern African countries. “Malaria Elimination 8 (E8)”. Available from: https://malariaelimination8.org/. Cited 29 Sept 2017.

[16] African Leaders Malaria Alliance. Available from:http://alma2030.org/. Cited 29 Sept 2017.

[17] Asia Pacific Leaders Malaria Alliance. Available from: http://www.aplma.org. Cited 29 Sept 2017.

[18] Evans DB, Etienne C. Health systems financing and the path to universal coverage. Bull World Health Organ. 2010;88(6):402 doi: 10.2471/BLT.10.078741 20539847 10.2471/BLT.10.078741 PMC2878164

[19] Rittel H, Webber M. Dilemmas in a General Theory of Planning. Policy Science. 1973;4:155–69.

