# Supplementary material for: malERA: An updated research agenda for health systems and policy research in malaria elimination and eradication
Source: PLoS Med. 2017 Nov 30;14(11):e1002454. doi: 10.1371/journal.pmed.1002454 (PMC5708613; doi:10.1371/journal.pmed.1002454)
Supplement: S1 Text — (DOCX) [file pmed.1002454.s001.docx]

S1 Text, supporting information file

For the figures and boxes referenced here please refer to the main manuscript.

**First step: Literature search**

To identify the relevant literature published since the initial malERA report, we searched electronic databases and abstracts books. The timeframe of the searches was January 1^st^ 2010 until May 18^th^ 2015, we did not apply any language limitations. Further details of the systematic search are described below. We did not access grey literature such as project reports, annual reports and government documents, as the focus was on the research that had been undertaken and rigorously evaluated (through peer review). The comprehensive literature search and synthesis provided the basis for launching the second step.

**Detail description of the literature review process**

Search terms used were:

("research agenda”) OR ("health management" ) OR ("information system*") OR ("service delivery" ) OR ("decision making" ) OR ("universal access" ) OR (planning ) OR ("implementation research" ) OR ("control and elimination" ) OR ("priority setting" ) OR ("integrated management" ) OR ("health system*" ) OR ("operational research" ) OR (“financing”) OR “equitable” [title, abstract] OR ("Health Planning"[Mesh] ) OR ("Health Services Research"[Mesh]) OR "Health Services/organization and administration"[Mesh]OR ( "Health Policy/organization and administration"[Mesh] OR "Health Policy/utilization"[Mesh])

AND (malaria) [title, abstract, Mesh] OR [list of LMIC countries in title, abstract] OR "Developing Countries"[Mesh] OR “developing countr*” OR (low and middle income countr*) OR LMIC [Title/Abstract]

We searched for indexed literature in the following electronic databases: MEDLINE (PubMed); the Cochrane Library, issue 5, 2015; Science Citation Index Expanded and Social Sciences Citation Index (Web of Science). We ran searches from January 1^st^ 2010 to May 18^th^ 2015, without applying language limits, and exported the search results to an Endnote database. We also searched abstract books of the Keystone Symposium 2014 (The science of malaria eradication), of the 63rd Annual Meeting of the American Society of Tropical Medicine and Hygiene, ASTMH 2013, and of the 6th Pan-African Malaria Conference 2013 (MIM). The final number of citations obtained, after de-duplication, was 4083.

The chairs of the panel further reviewed the abstracts of all papers identified by the search and removed all references linked to: critical care/radiotherapy/veteran’s health; clinical trials; Willingness to pay studies; RCTs of drugs/therapies/diagnostics (keep in ones of programmes, community participation, health promotion, health workers programmes, health systems);   Epidemiological studies; Any studies on immunity ; Cost effectiveness studies except for malaria; Genomics; Technical vaccine related; Legal frameworks unless linked to migrant/mobile health/services or counterfeit drugs; Environmental issues other than linked to malaria ( eg. marine biodiversity); Mental health programmes and Most HIV unless  it is about service/programme management/coverage of services/health worker related; as they were not relevant to the topics of the panel.

The articles were then reviewed in-depth by the chairs and only 41 papers were found to be relevant to the theme of health systems and policy research in the context of malaria pre-elimination, elimination or prevention of re-introduction. These were then made available to all panel members, who were also invited to identify their top papers relevant to theme if they have been missed by this process. This did not add anything to the pool.

Briefly an analysis of the final 41 papers found that on the whole they continued to re-iterate the gaps and barriers identified at the time of the 2011 malERA activity. Very few of the papers discussed actual interventions, the others identified barriers or gaps but did not provide solutions nor interventions to develop. A few papers described a project being planned or started, but no outcomes yet published, even if the original paper was from 2011-12.

Only 10 explicitly mentioned elimination and only three were specifically in pre-elimination or elimination settings. The regional distribution of the papers (some have more than one region) were: 15 from Asia (mainly China and India), 27 from Africa, 2 from Latin America, 1 from the Pacific and 7 that were generically global. The interventions included: integrated elimination package in China (Zhu et al 2014); adding Intermittent preventive treatment in infants (IPTi) into routine health services (Akoria et al 2014), adding bed net distribution to a agricultural loan programme (Fink 2012); ways to improve the quality and outcomes of training on RDT introduction and cost analysis (Kyabayinzi 2012); participatory health education in schools as a pilot (Ayi 2011) .

Based on the Panel’s analysis of the literature the common issues discussed in the papers were: Financing including vouchers, national health insurance and Global Fund; Health information systems including monitoring and evaluation and use of data, programme evaluation, spatial decision support systems, and surveillance and response; Human resources including training of health staff, type of staff; Commodities drug management and logistics; Governance : 1 paper on drug policy; Service delivery : integration, private sector retailers, cost effectiveness of approaches; and some generic papers on status of programmes and trends.

**Second step: Panel approach**

A two day workshop was held with the majority of the panel members. The panel broke into small working groups to identify the health systems issues that needed to be addressed in malaria elimination, based on the literature and their own experiences. Each one of these was placed on a card for later priority setting activities. To organise the health systems research needs in malaria elimination, the panel adapted the matrix organization from the initial malERA process. The malERA Refresh matrix uses three dimensions: health system building blocks; health systems levels; and research categories (Fig 1). Six health system building blocks described in the health “Health system thinking” framework by the Alliance for Health Policy and System Research were adopted here [1]. The research questions were classified as falling under community, facility or district, national, regional/ global levels or being pertinent to all levels. Research questions have been grouped in the three categories (Box 2): Evaluation science; Research; and R&D. This kind of portfolio approach should make it easier for researchers and funding agencies to identify their subjects of interest.

The country groupings used in the previous malERA paper (i.e. group 1 (very large malaria burden where malaria elimination is considered impossible with existing tools), group 2 (countries with focal malaria where interruption of transmission is deemed possible) or group 3 (countries that are elimination-ready) were abandoned. The panel adopted the stance described in GTS and AIM whereby malaria prevention and control efforts move along a continuum without clear-cut categories and that many countries are a blend of settings along that continuum.

Each of the “cards” with a topic, gap, need or question from the small group activities was reviewed by the panel as a whole to be written into a research question format. The panel broke into smaller working groups to identify whether these topics needed research (R), needed tools/intervention developed and trialled (R&D), or could be addressed through evaluation (E), or a combination of these. Each group then refined the research question. Each group fed back to plenary where further robust discussions and input occurred. This helped finalise the uber-matrix of research questions across system levels, building blocks/cross cutting themes and research category (R, R & D or E).

Finally, the panel, used the modified Nominal Group Approach [2] to identify the top two priority research questions in each of the research categories. This approach allows panel members to place additional emphasis on a question of their choice that can be similar to or different from the top 2 listed above. This process was iterative asking each participant of the panel meeting to identify his/her top picks followed by a wider consultation of the other members not physically present to follow the same process.

The outcome of this workshop was shared after the meeting with all panel members, and through an iterative process finalised.

**References**

1. Research WAfHPaS. Systems Thinking for Health Systems Strengthening. 2009.

2. Delbecq AL VdVA. A Group Process Model for Problem Identification and Program Planning. The Journal of Applied Behavioral Science 1971;7:466-92.
